# Being a Surrogate Partner: The Challenges of Fragile Boundaries

**DOI:** 10.1007/s10508-024-02821-9

**Published:** 2024-03-06

**Authors:** Ayelet Oreg, Elad Avlagon, Tamar Gitlitz

**Affiliations:** 1 The Louis and Gabi Weisfeld School of Social Work, Bar-Ilan University, Ramat Gan, 5290002 Israel; 2 Sapir Academic College, Hof Ashkelon, Israel; 3 The Bob Shapell School of Social Work, Ramat Aviv, Tel Aviv, Israel

**Keywords:** Surrogate partner therapy, Boundaries, Sex therapy, Identity, Intimate surrogate partner

## Abstract

Surrogate partner therapy is a type of treatment in which the surrogate partner (SP) works in a triadic setting with a sex therapist and a patient. At the same time, the SP acts as an intimate surrogate partner to the patient. The SP treatment includes a range of therapeutic experiences such as relaxation, intimate communication, sensual and sexual contact, and training for the acquisition of social skills. In the current study, we ask what and how SPs experience, understand, and construct boundaries in their work. We used Winnicott’s therapeutic conceptualization of psychotherapy as a mode of playing and Goffman's dramaturgical role theory as the theoretical framework for our exploration. Applying a phenomenological and empathic approach, we analyzed 13 in-depth interviews with Israeli SP. It appears that SP’s transitions from one performance to another are dramatic, in that their role requires the involvement of sexual and emotional helping relations with their patients. Moreover, SPs are obliged to have secrecy at all levels and in various relationships in their lives. We uncovered various complexities that SPs experience, such as a lack of clarity about their role, which creates challenges for building their professional and personal identity and affects their family and social relationships.

## Introduction

Surrogate partner therapy (SPT) is a type of treatment in which the surrogate partner (SP) works in a triadic setting with a sex therapist and a patient (Aloni & Heruti, 2009). The treatment is for limited period, during which the SP acts as an intimate partner to the patient and includes a range of therapeutic experiences, such as relaxation, intimate communication, sensual and sexual contact, and training for the acquisition of social skills (International Professional Surrogates Association [IPSA], 2023). The goal of SPT treatments is to help patients to raise self-awareness and acquire skills in the areas of emotions and intimacy. In this challenging therapeutic setting, SPs are required to perform their work within various dynamic boundaries. Whereas ample research has looked into the experiences of SP clients (e.g., Aloni et al., 1994, 2007), very little is known about the experiences of the SPs themselves.

In this preliminary, exploratory, phenomenological study, we ask what and how SPs experience, understand and construct boundaries in their work. We used Winnicott's (1991) therapeutic conceptualization of psychotherapy as a mode of playing and Goffman's (1959) dramaturgical role theory as the theoretical framework for our exploration.

### Sex in Research and Therapy

Human sexuality is a complex phenomenon, involving the interaction of one’s biological sex, core gender identity (sense of maleness, femaleness or other gender identity), and gender role behavior (nonsexual as well as sexual). These factors combine with values, attitudes, feelings, interactions, and behaviors and impact the manner in which an individual expresses their sexuality emotionally, socially, culturally, and physically (Merrick & Greydanus, 2016). Sex is demonstrated as an important aspect of western societies through portrayal in media, advertisements, and education (Meston & Buss, 2007). The reasons people turn to sexual therapy vary. The most commonly stated reason is a lack of satisfaction from their sex life, which affects their mental and physical well-being (Seitz et al., 2020). Furthermore, in a large survey conducted by Mitchell et al. (2016), 38.2% of sexually active men and 22.8% women reported experiencing sexual dysfunctions. Sexual dysfunctions are a source of mental distress, anxiety, depression, low body image, sense of insecurity and low self-worth, as well as other issues. Moreover, these issues can damage relationships between partners, and often cause difficulties in daily functioning (Militscher, 2020). In addition, the level of a person’s sexual activity and functioning has been determined to be a strong predictor of longevity and has great importance regarding its sexual health and well-being (Flynn et al., 2016; Killingsworth & Gilbert, 2010).

Previously, both therapists and people perceived sex therapy as part of treatments in the context of procreation (Bechthold, 2020). Later, it became a separate distinct area of specialization as scientific sexuality research became more widespread. Kinsey et al.’s (1953) research extensively presented various sexual behaviors and tendencies, that were considered taboo until then. This research initiated public and professional discussions and was the beginning of legitimization for engaging in research and discourse about sexuality (Herzog, 2006).

Following this, in the 1960s, therapy methods known today as sexual therapy were developed for the first time, included physical and mental exercises, and constituted for the first time a separate distinct field of research and therapy (Apfelbaum, 1977; Hartman & Fithian, 1972; Kaplan, 1988; Kinsey et al., 1948). Masters and Johnson were the most influential for this evolution, following their 1970's *Human Sexual Inadequacy*, which developed the notion that sex therapy as a special form of therapy requiring highly specialized training (Binik & Meana, 2009; Hartman & Fithian, 1972; Kaplan et al., 1982; Masters & Johnson, 1970).

Current treatment methods for perceived sexual concerns commonly involve individual or couple psychotherapy, potentially combined with medical interventions. Within psychotherapy, treatment commonly includes processing thoughts and practicing different exercises or tasks between sessions, such as practice of communication skills, sex education, sensate focus, systematic desensitization, masturbation, mindfulness-based Intervention for women, "squeeze" methods for males (Alahverdi et al., 2022; Albert et al., 1980; Atallah et al., 2016; Gottman, 1999; Kilmann et al., 1986). Moreover, medical interventions may include Viagra/Cialis, which assist with erectile dysfunction—the consistent inability to achieve or maintain an erection that is sufficient for satisfactory sexual intercourse (Burnett et al., 2018; Sadovsky et al., 2011; Shamloul & Ghanem, 2013).

### Surrogate Partner Therapy

SPT is a form of therapy developed as a result of the work of Masters and Johnson, who tried to find solutions for the sexual needs of patients who were not part of a marital system, therefore could not be provided with the classic couples therapy as was available prior to that time (Aloni & Heruti, 2009). Concerns that lead patients to seek SPT might include negative body image, physical disfigurement, fear and avoidance of sexual and emotional intimacy, lack of relationship experience, lack of social or sexual self-confidence, etc. (International Professional Surrogate Association [IPSA], 2023). Specifically, common sexual issues for male clients include erection difficulties and ejaculatory inhibition, while females seeking SPT might suffer from vaginismus (involuntary contraction of vaginal muscles resulting in painful penetration), shame or anxiety regarding sex etc. (IPSA, 2023).

SPT is a treatment method that is used in Israel, Great Britain, Australia, Germany, the Netherlands, and the USA, and adapted in accordance with local culture and laws (R. Aloni, personal communication, February 28, 2023). The IPSA operates as a prominent professional association for surrogate partners in the USA with voluntary membership that includes the obligation to adhere to the IPSA code of ethics, although there are no legal consequences for practicing SPT without IPSA membership (Bechthold, 2020; IPSA, 2023). The course of treatment starts with a full diagnosis conducted by a clinician and a doctor, who determine the type and goals of the therapy together with the patient (Aloni et al., 2007). If SPT is chosen for the patient, a detailed explanation regarding the process is given, and a surrogate that is potentially suitable for him/her is chosen in a mutual agreement (R. Aloni, personal communication, February 28, 2023).

An introductory meeting is then held between the surrogate partner and the patient, after which both can agree to continue or to cease the interaction. If one of them refuses to continue, the surrogate is replaced. However, if there is a mutual agreement to continue, medical testing (for sexual transmitted diseases, and vaccination against hepatitis B) is conducted for both surrogates and clients prior to the first session and every 3 months thereafter. In addition, both parties sign commitments to practice safe sex during therapy and in outside relationships (R. Aloni, personal communication, February 28, 2023). The therapy is conducted according to the therapeutic procedure, both in a dating environment that is social (e.g., in a coffee shop), and in a specialized apartment-like setting in the clinic. This area contains a room with a foldout sofa that opens into a double bed if needed, a bed linen box, and a bookcase where there are required accessories, such as gel, body lotion, condoms, and accessories for sensate focus (feather, sponge, velvet, rough fabric, etc.).

Moreover, SPT is considered short-term treatment, and usually lasts for several months of weekly encounters between each part of the triad.^1^ That is, sessions with the therapist are interwoven with the surrogate/client sessions, following the understanding that there will be open communication between the three members of the triad (Poelzl, 2022). The therapy with the therapist can be seen as a more “theoretical” session, while the SP sessions are the “practical” implementations of the various social and sexual skills practices (e.g. masturbation, penetration) (Aloni & Heruti, 2009). The main goal is to prepare the patient for relationships; therefore, everything that can happen in a relationship between partners is part of the treatment, including expectations, disagreements, silence and misunderstandings.^2^ Accordingly, both sides should expect to experience real emotions, as well as develop social aspects and attachment as part of a complex and dynamic intervention involving touch (Bechthold, 2020). The surrogate partner consistently reports back to the sex therapist on the therapy's progress. That is, the therapy is conducted in a manner that enables the therapist to address the needs of both the client and the SP (Rosenbaum et al., 2014). Ultimately, when it is agreed that the therapeutic goals in the surrogate-patient sessions have been achieved, the patient separates from their surrogate partner, and continues the treatment with their therapist. The purpose of this stage is to examine whether the therapeutic needs were met, focusing on making the necessary transition to regular partners, whom they will meet in the natural circumstances of life (R. Aloni, personal communication, February 28, 2023).

### Therapeutic Boundaries: Donald Winnicott and Erving Goffman

Winnicott defined psychotherapy as a "potential space" where two subjects play. This play is a way of communication between the subject and him/herself, and between "me-extensions and the not-me," which includes spontaneous connection to the "true self,” to thoughts, memories and associations (Winnicott, 1991). Moreover, psychotherapy enables a mental dimension relating simultaneously to the patient’s inner and outer reality, but also creating the potential space between reality and fantasy. This space creates a paradoxical reality which is used as the framework of the treatment and creates the unique quality of the relationship established during it. The possibility of maintaining the paradoxical relationship lies in the "understanding" of this reality as well as a result of the therapeutic setting (Yakobov-Ivry, 2009).

The therapeutic setting includes a complex relationship, which Leigh and Silbert (2016) well described as a "relationship that requires deep attachment, regression, and the fulfillment of deficit needs—all this, inside a professional relationship that simultaneously requires boundaries, limits, and the valuing of autonomy" (p. 328). Thus, the therapist is required to work within a set of rules that distinguish this from other relationships, differentiating their reactions to the client's instinctual wishes and ego needs—the former being more aligned with description of basic drive needs, while the latter being the needs that are connected to the development of the self (Mitchell, 1993).

The unique role required of SP can also be analyzed using the dramaturgical role theory proposed by Goffman. First of all, it is important to note that Goffman has been negatively framed as a sociologist who had no interest in the interiority of human experience. However, on the contrary, he expressed genuine and profound interest in psychoanalytic themes, such as unease in interpersonal relationships, regulation and transgression, and the textual and performed qualities of the self (Goffman, 1959; Hancock & Garner, 2015). Furthermore, Goffman claimed that social interaction can be metaphorically explained in terms of theatrical performance. That is, when the individual is in the immediate presence of others, his activity will have a promissory character that he performs, based on cultural values, norms, and beliefs (Goffman, 1959).

This capacity appears to involve two radically different kinds of activity: the expressions that the individual gives, and the ones he gives off (Goffman, 1959). The first involves verbal symbols or their substitutes, which he uses to convey the information that he and society are known to attach to these symbols; Goffman called this the "front stage.” One the other hand, the "back stage" is where the “actors” is required to adjust their identity as part of their interactions with the "audience,” before “entering” the front stage: i.e., cultural values, norms and beliefs with which they are expected to correspond accordingly (Quist-Adade, 2019).

Our standpoint in exploring the SP’s work, is through Goffman’s theory and the understanding that it can applied to the different "personas" and performances that SPs are required to negotiate in different settings in their lives (both professional and personal lives), creating boundaries within themselves and with their outer world. This is an internal process that evolves for each SP during each and every relationship. Thus, as noted above, our aim in this study is to understand what and how SPs experience, understand and construct boundaries in their work.

## Method

We adopted a qualitative, descriptive phenomenological approach, involving two distinct features. The first is the aim to reach a deep understanding of each participant's individual experience. The second is to go beyond individual descriptions of various experiences to “disclose the essences, or structures of meaning immanent in human experiences” (Finlay, 2012, p. 7). We chose this approach based on the premise that individuals’ identities and their perceptions of reality are socially constructed. The unit of analysis was Israeli SPs, all employed at Dr. Ronit Aloni's clinic. All SPs who participated in this study are required to follow the same practices and guidelines.

### Study Context and Research Ethics

The experiences of SPs in this study are situated within the Israeli sociocultural context. Sexual surrogacy in Israel is a legal and accepted practice, meeting IPSA’s strict standards of conduct (Rosenbaum et al., 2014). Most of the surrogacy services in Israel are provided at Dr. Ronit Aloni's clinic. The clinic was established in 1996 as a multidisciplinary center, with the view that surrogate therapy is an integral part of the rehabilitation process (Aloni et al., 1994; Yakobov-ivry, 2009). The clinic upholds surrogate training standards by providing guidance on recommended practices, legal issues, and medical procedures to mitigate health concerns related to sexual contact, and surrogate training standards (Bechthold, 2020). The prevalence of surrogate therapy worldwide is unclear and appears to vary with geographical location, partially because of legal ambiguities. According to Aloni (2023), since the clinic’s founding in 1996, more than 500 women and men have undergone sexual therapy with the help of a SP in Aloni’s clinic and several more in other clinics. Approximately 65% of clients were men. SPT has been approved and is partially funded by Israel’s Ministry of Defense for military veterans with various disabilities (e.g., spinal cord and brain injuries). Given the lack of separation between state and religion in Israel, SPT in Israel is unique in that only unmarried individuals are legally permitted to become surrogate partners.

According to R. Aloni (personal communication, February 28, 2023), the training process for surrogate partners in her clinic begins with a screening process, to ensure the candidate is suitable for participation in the course. After passing the screening, surrogates receive approximately 40 h of training, including two workshops, lectures by sexual therapists that provide basic knowledge about sexual therapy, and case studies presented by a trained SP. At the end of the course, trainees are required to complete a sociometric questionnaire and are evaluated by the course lecturers. Aloni and her staff then compile the data and select the trainees that appear to be ready for work in the clinic. These go through an external evaluation to verify that the trainees do not suffer from untreated sexual trauma and that they have the necessary skills (e.g., the ability to set and maintain boundaries) for work as an SP. New SPs first receive one patient, and Aloni serves as the sex therapist in the triad, who holds weekly supervision sessions throughout the treatment.

### Participants

Participants for the study were recruited at one of the clinic’s staff meetings, in which we introduced ourselves and described our research questions. Twelve SPs volunteered to the study, in addition to Ronit Aloni, the director and founder of the Israeli SPT clinic (see Table 1 for sample member characteristics). We conducted in-depth interviews with all 13 of them. The third author conducted the interviews via Zoom, which were later transcribed. Each interview lasted between 60 and 90 min (see Appendix 1 for sample interview questions).

**Table 1:** Participants’ demographics

Pseudonym	Sex	Age	Number of years of working as a surrogate	Family status	Additional profession	Type of relationship Monogamous/non-monogamous
Annie	Female	57	13 years	Single	Teacher	Monogamous relationships—on and off
Sarah	Female	40	10 years	Single	CBT therapist	Non-monogamous
Dor	Male	41	11 years	Single	Masseur, mindfulness, natural language processing & Tantra instructor	Non-monogamous
Magen	Female	37	10 months	Single	School counselor	Non-monogamous
Anat	Female	45	5 years	Single, in a relationship		Non-monogamous
Inbar	Female	39	2 years	Divorced		Non-monogamous
Shelly	Female	47	1 year	Divorced	Teacher	Non-monogamous
Maya	Female	35	14 months	Single	Project manager	Not in a relationship
Eden	Female	43	1 year	Married, in an open marriage	Kindergarten teacher	Non-monogamous
Idan	Male	45	9 years	Single, in an open relationship	Musician and artist	Non-monogamous
Shalom	Male	46	6 years	Divorced, in a relationship	Hi-tech	Monogamous
Nitzan	Female	35	7 years	Single	Musician	Not in a relationship

### Analyses

We conducted a thematic analysis using a phenomenological approach, seeking both commonalities and differences among SPs’ experiences. Throughout this analysis, our emphasis was on the SPs’ descriptions of their experiences (Finlay, 2011). Our analysis yielded descriptions, from which we identified themes, and interpretations. For example, content such as using Viagra, shaving legs, putting on perfume, and using nail polish were assigned to a theme we labeled “body transitions from the outside world to the treatment room.” Content pertaining to SPs’ use of pseudonyms with clients, and mentions family members, friends, and colleagues not knowing about the treatment were coded into a “secrets in the SPs’ lives” theme. All three authors coded the transcribed interviews. First, each coded the text individually. Next, we held several meetings to discuss each interview and to create a code book. We then recoded all of the interviews and finalized the themes that best suited our research question.

## Results

Through their interactions with their clients, the sex therapists, and their personal families and partners, we illustrate the SPs’ challenges in setting boundaries. Goffman (1959) suggested that we adopt diverse personas and enact distinct roles that are expected of us, shifting from our genuine selves in “the backstage,” and those socially defined roles in the public stage. For example, a pesona suitable for the parent–child relationship may not be suitable for one’s work setting. From our findings, it is apparent that SPs navigate multiple personas, transitioning between different “stages” and adopting a variety of roles. These transitions occur in SPs personal and social spheres, encompassing various responsibilities, including those in their families and their professions outside their work as SPs. Moreover, findings shed light on the clash between a conservative discourse and a post-modern discourse related to sexuality, relationships, and family ties in Israeli society. We suggest that such a clash makes it harder for the SPs to navigate their different personas and roles. We use the notions of “persona” and “performance” by Goffman (1959) to examine the diverse identities and roles assumed by SPs within their work, with their families, and in their social lives beyond the clinic. In persona we refer to one's personality as manifested towards others, as in one’s social role (Goffman, 1959). In our analysis, SPs’ various personas serve as a deliberate identity masks in public, and in the treatment room. We use the terms "identity" and "persona" loosely, encompassing impressions of oneself and social behaviors in SPs’ lives.

Overall, we identified five themes:

1. Surrogates’ role perceptions: Professional boundaries
2. Who helps the surrogates? The framework for training and retention in the position
3. “As a surrogate I am in character, at some level or another”: bodily and emotional boundaries in surrogate work
  (3.1) The performance of the body: when the body doesn’t cooperate
  (3.2) The blurred boundary between partner and client
4. “There’s a difference between sharing and hiding. The fact that no one knows what color my underwear is, that’s not because I hide it, it’s because it’s not their business, ok?”: family and social boundaries in surrogate work,
5. Boundaries from a social-cultural perspective

(1) Surrogates’ role perceptions: manifestations of professional boundaries in surrogate work

Many interviewees mentioned the regular setting, bounded by time and place, which includes a prohibition on maintaining contact outside the therapeutic meeting, as an anchor that allows an easier transition from the world outside the therapeutic-surrogate relationship, to the world within. According to them, the rigid framework provides security within the position and freedom to be completely present with the patient.

I think that [this] is a great advantage, beyond the fact that we meet once a week, there is no interaction, for me this is really suitable…we have our hour and a half, we are both so focuse…we come to devour this hour and a half, I feel this, let’s say, something that greatly helps this to succeed […] I met him seven times, it’s like I don’t like know him like I would know someone who I would become their partner. It’s to come there for enjoyment…we simply come focused on the partnering between us, mainly the touch. (Eden)

Eden's quote raises questions regarding their role as SP, for example, is it indeed, the goal of this treatment “to come there for enjoyment”? who’s goal is it? does it represent the goal of the patient? or the goal of the SP? Another instance of boundaries can be found in the question of to what extent the surrogate, in the next case a man, is, in his role, a "service provider," can or should absorb criticisms or offensive statements from the client. Since this is a relationship that should resemble a marital system that includes intimacy and security, there is difficulty in deciding when and if one should set limits and end the relationship if the limit is not respected. In Goffman's theoretical terms—when may the surrogate "take off" the surrogate persona and decide to stop playing the role of the partner in order to protect their own "self.”^3^

I had a client who was 47 years old, who came to treatment in order to reach orgasm. It was very, very difficult with this client. I felt that whatever I did, she was very critical and demanding, and even at the energetic level, it was very difficult for me with her. She would say very insulting and hurtful things…It is difficult to create intimacy with someone like this, and as much as I tried to break through her defensive walls, and as much as the [sex] therapist tried, I felt that it was not going anywhere and, in the end, I requested to stop the treatment. It was the only time in my life I did that. (Idan)

The following quote contains a variety of challenges:

You must have emotional and empathetic ability at a very, very, very, very high level, very, really, for very fine subtleties of sensitivity, ah, as if this comes naturally to you…And on the other hand, to be able to have boundaries for your personal life, as if, to make separations. It is really total dedication without any inhibitions. (Sarah)

Sarah’s words present the difficulty and complexity that all the interviewees emphasized: on the one hand, the clear boundaries and separation between private and professional life, and on the other hand, the emotional devotion described as "uninhibited.” Thus, the role experience is characterized as holding the two contrasts of clear boundaries ("making separations") in the face of boundless total dedication. In this case, Sarah describes that this transition "seems to come naturally,” which perhaps can also shed light on the uniqueness of the profession, and the characteristics of those involved in it. The word "as if" can be interpreted in two ways—in simple terms, "as if" is also a slang word, and as such is just a style of speech. But in this statement, it can point to a deeper difficulty—so that maybe it is not so simple or natural (or possible at all?), to surrender totally.

(2) Who helps the surrogates? The framework for training and retention in the position

As part of discussing their professional boundaries, the surrogates also chose to refer to the training and maintenance framework of their work, which includes several work interfaces beyond the meeting with the patient—one includes training together with the sex therapist and the clinic manager, and the other deals with the patient and includes the sex therapist and the meeting coordinator. The transitions between these interfaces require behavior from the surrogates that is sometimes perceived as complex and not clear enough. In addition, questions arose about preserving confidentiality—what is the significance of the introduction of the sex therapist as an additional player in the therapeutic relationship system? In Goffman's terms of persona and play, there is an exit from the playful space between the client and the surrogate. There is an entry into another space between the surrogate and the sex therapist, a space where the "partner" becomes the therapeutic object.

In sexual contact you, you are not completely, you are not alone there. That is, the therapist is there with us in spirit. She doesn’t, she doesn’t watch us of course, but she is there with us in spirit and after this you need, the fact is that you need after this to document and to describe everything you felt, everything you felt, everything that happened, it partially takes away the…from the sexual contact its—its pure moment, the connection… (Idan)

Magen elaborates on the challenges regarding the unique supervision setting:

So the supervision [takes place] with the [sex] therapist, with the clinic director and me. Also, there is a second supervision which includes the client, me, the sex therapist and the meeting coordinator. So it’s as if there are several work interfaces…Moreover, if a client raises a difficulty while in a session with me, about his sex therapist—where is my place in it?, because if I am now in a supervision setting, with the director of the clinic and the sex therapist [and I know something that the client didn’t shares] Should I share this info? this triangle is complex… (Magen)

(3) “As a surrogate I am in character, at some level or another”: manifestations of bodily and emotional boundaries in surrogate work

In their work, surrogates are required to use a variety of skills, both physically through the body and on the emotional level. This is to establish communication and a connection with the client. In the interviews, the surrogates referred to various cases in which the body and emotion did not correspond with each other. During the interviews, surrogates shared the various complexities related to the body being the "working tool" in therapy. The connection between body and emotion is always challenging for surrogates. There are situations in the treatment room where the body "refuses" to cooperate. The current section reveals different instances of bodily and emotional challenges during the surrogates' work.

(3.1) Bodily performance: the body doesn’t cooperate

In terms of the mind–body connection and bodily performance—it is possible to explain and interpret the various experiences of the interviewees as a continuum, on one side of which there is an experience of body and soul connected together in a holistic experience, compared to the other end of a continuum—where the sexual bodily experience is separated or disconnected from the emotional experience. It seems that the surrogates move on this axis between full connection during the sexual experience and disconnection. Whereas two interviewees described either end of the continuum, the rest of the interviewees described moving between the two poles. Another surrogate described a complete connection between body and mind during treatment with a certain client. However, she stated that this does not mean that she will have the same experience again, and moreover, even during contact with one client there may be "shifts" on this continuum.

…As the treatment progressed, I already felt how my body began to close up…It’s hard for me to put my body into this whole story. That is, I can be there for them forever with my heart, but it’s hard for me to be there with my body, that is, it’s hard for me that my body is, is a therapeutic tool. My body simply doesn’t, doesn’t open up…but when we’ve already gotten into the whole business of, of getting undressed and sexuality and…my body doesn’t, it’s hard for it to open up (Maya)

When Maya performs in a sex-therapeutic relationship, the body sets a clear limit for its performance and refuses to conform. On the emotional level, there is a "boundless" performance ("I can be there for them forever with my heart"), but on the physical level, the body sets a clear limit and refuses to engage in the relationship. In Maya's case, after several unsuccessful attempts to continue in the position, she retired.

Maya's quote about the body being a "tool in therapy" is an experience repeated by all the interviewees. All of them described at least one or more cases in which their body and emotions were not in harmony. However, there was a large difference among the interviewees regarding the frequency of the phenomenon and the way it was experienced.

As mentioned, in the interviews we conducted with 12 surrogates, most described their engagement on a continuum. Only one described that her body and emotions are always in harmony, at the opposite end of the spectrum was Maya, who chose to leave the profession because she was unable to deal with this emotional-physical complexity. The rest of the interviewees moved on the continuum between connection and disconnection between body and emotions. Each described how they dealt with this challenge. The men we interviewed raised an unusual difficulty—what happens when the body does not cooperate and erectile dysfunction interferes with the treatment process?

…Sometimes we male surrogates have this difficulty that female surrogates do not have, that you, you can’t lie, lie with your body. That is, you can love someone very much and you can relate to her very much and want what’s best for her, and it still, something there for all kinds of reasons doesn’t work, and you sometimes need extra help, and that’s ok… (Idan)

So what’s preferable? To take Viagra? Not to take Viagra?…On the one hand, we are there for her, so if we came to practice penetration, then it’s preferable to take Viagra, otherwise there won’t be, there won’t be an erection, most likely, and if, but if there’s no erection that’s a situation that she might encounter in the real world, so maybe yes give her the opportunity to cope with this kind of situation that her partner can’t get it up. (Dor)

Another boundary can be found in the clear directive about condom use. As mentioned, these are the clinic rules, which all patients and surrogates sign when starting treatment. The following interviewee referred to the issue of condoms as a directive that can make it difficult to connect body and emotions in the treatment room:

Sometimes it’s the tension of the room and the need to use a condom…it takes me some time to get things started […] and sometimes we need to get help from Viagra […] like, if you can take something that makes it easier for you, it doesn’t mean anything about your functioning as a man. It means that in a constellation that is so stressed and ense, if you can take something that can help you physically, then why not? Absolutely… (Idan)

Some of the interviewees referred to emotional and physical preparation before entering the treatment room. Preparation is a "rite of passage" that prepares the body and emotions for the treatment session. In Goffman's terms, moving from role to role, putting on a persona, and entering the “front of the theater stage” of the therapeutic relationship. The following quote describes it well:

It's very pleasant for me to step out of my routine, I have a date once a week, he is a man for whom make-up is very important and I am the least made up woman in the world, so I am suddenly like putting on nail polish that he requested, and I really make an effort to remove bodily hair, which is something that before, I really didn’t care about, and my partners didn’t either…he bought me perfume, I am like really a kind of a different persona, and it’s very pleasant for me this game/act, as if it brings something else out in me, which is still me, but something different that I really desire would be externalized… (Eden)

(3.2) The (blurred?) boundary between partner and client

…I had one client I fell in love with, I really fell in love with her, because it was as if…in the sense that she knew that I would not violate any boundary or anything like that, but I allowed myself truly to feel it in all 100 percent, because she was truly, she was an amazing young woman… (Dor, a male SP)

During the interviews, the participants were asked about the difference in their experience of relationships with partners in everyday life versus the relationship between the client and partner within the treatment room. Different behaviors emerged from the answers indicating a different experience of contact. For example, one interviewee stated that in her role experience, there was almost no difference between a romantic partner and a patient, so she allowed herself to behave freely in the treatment room, for example, she ate during the treatment and shared with the client that she was menstruating and therefore did not wish to have sex. Her choice regarding self-presentation can be interpreted as blurred role boundaries (partner-patient). In contrast, other interviewees emphasized clear boundaries at the level of behavior—such that the treatment room (In Winnicott’s term, "the play space") determined the staging and the rules of work and relationships, which helped preserve the difference between their experiences of their actual partners and their experience of their treatment partners. In the emotional world, it was apparent that it is more difficult to separate, and interviewees shared a variety of emotional experiences that characterize romantic-couple relationships. For example, falling in love, desire, or alternatively the lack of sexual attraction that led to the question "how far to play the game" or simply not accepting a patient for whom they did not have sexual attraction. This can be seen in the following quote:

I am not sure, let’s say, that I would take any client. For example, they suggested a client for me who was older than me by almost 20 years, and it seemed like this was too big for me, and that it would be difficult for me to connect, so I said that I would not be willing. (Shalom)

As with many meaningful relationships, both therapeutic and non-therapeutic, breakups are painful and charged. From the following description of the breakup, one can learn about the complexity of the transition from the treatment room to the world outside it. This is when the rules of ethics construct a clear boundary that does not allow "leakage" of the treatment relationship into a romantic relationship in the world outside.

…there were situations of falling in love, really falling in love, and it was so hard to separate, really, really it was hard to separate. We said afterwards [e.g. after the last meeting] to Ronit, the last meeting lasted three hours, ‘what can I do? I couldn’t do it’. I, not that I took his telephone number and was in touch with him, right? I didn’t do something forbidden, but it was with a lot of tears and pain… (Sarah)

In conclusion, during the interviews, the topic of falling in love between surrogates and clients and vice versa came up. The current study deals with the surrogates' experiences and not the experiences of the clients. We chose not to explore the complexity related to content and theoretical worlds related to clients falling in love with therapists. Instead, we focused only on the surrogates. Since this topic deals with the emotional and behavioral relationships between patients and surrogates, and the issue is how similar or different the treatment is to another couple relationship, it was important for us to mention the "other side" of this relationship, as it is reflected in the words of some of the interviewees, for example:

Usually, there are always instances of falling in love in treatment, but usually it’s from the side of the client toward the surrogate. (Dor)

Thus far, we have defined boundaries within the surrogates' work. In the next section, we will focus on the boundaries surrogates enact at the level of family, society, and culture.

(4) “There’s a difference between sharing and hiding. The fact that no one knows what color my underwear is, that’s not because I hide it, it’s because it’s not their business, ok?” Family and social boundaries in surrogate work

In the employment contracts for surrogates in Israel, they are required not to live in an institutionalized relationship. Thus, every surrogate must be single or divorced. This is done to avoid lawsuits and in addition, there is a halachic aspect (Jewish religious law) to this requirement. The interviews show that all the interviewees are in non-conforming relationships with Israeli society [open marriages, non-monogamous relationships, polyamory, etc.] or are single. For example, Inbar, who married in her youth after a long relationship with her partner, describes that after several years of a monogamous relationship, and children together, she and her partner chose to open the marriage and experiment with sex and relationships with other partners—in the case of her partner, also partners of the same sex. She describes:

Currently we have a home, we have a daughter in common, we are raising my two older children together, so today it doesn’t make a difference to me if he sleeps with men, sleeps with women, if he falls in love with whomever he wants, we still have a home her to run and our connection is not, not something that it is possible to simply cut off easily…and I managed to connect to this very quickly, like, to recognize the new conditions and to get along well with them. So it basically gave me the initial sense of hey, maybe I would also be a good surrogate… (Inbar)

I am in an open relationship with a married woman, I live alone, throughout the years as a surrogate I almost always had relationships simultaneously, of course I always told my partners what I work in, and because I come a bit from these worlds of freedom and very liberal spaces like this so mostly I did not have a problem… (Idan)

Another complex issue is the ability of the surrogate’s partner to be supportive of their occupation. Indeed, several interviewees shared that they hid being a surrogate from their partners, at least at the beginning of the relationship. One of them even mentioned that her partner supported her work as a surrogate at the beginning of the relationship, but it became too difficult for him and he left. From the life choices of the interviewees regarding their own relationships, it is possible to learn that they have unique abilities to contain and conduct themselves within more flexible boundaries, feeling held within them. Eden also shared the relationship she maintains concurrent with her work as a surrogate:

I feel that [my work as] a surrogate does not challenge my partner at all […] He is completely aware of this, and he said that he also feels very great pride that I do this, he really feels lucky that he lives with a woman for whom it is comfortable to do this and who does it with such love and ease… (Eden)

In the interviews, the issue of hiding one's occupation as a surrogate for family members and friends came up. Some of the interviewees described frustration that they have powerful experiences within their occupation, but cannot share them with the significant others in their lives.

…my parents, from my point of view, I put on a mask every time I go to meet them and every time I speak to them on the telephone…to my regret this is the only place in my life that I behave this way, but I simply have no choice because they will never accept me as I am. My close friends know…if it was up to me all my friends would know, because I’m not ashamed of this… the limitations that I place on myself in this matter exist not because of my personal will, but simply because of the matter of confidentiality… (Anat)

(5) Manifestations of boundaries from a social-cultural perspective

Surrogate work produces a tense encounter between two social discourses concerning sexuality and sex (e.g., the conservative discourse vs. the neoliberal one). Some of the interviewees frequently used the Hebrew word ‘כאילו’ that can be understood as the Hebrew Slang for “like” or “kind of” whenever they described the possible connection between SP and prostitution, a comparison that raises tension and discomfort among the interviewees. It may be that this comparison resonates in the consciousness of the surrogates and accompanies them during their work.

…And also vis-a-vis myself, it’s as if, yes, there is sometimes the, the thought, this parallel to like sex services, it is clear to me that this is not the same thing… there is in this like, sort of an element a bit like prostitution, because I am also paid for this that I sleep with him… (Magen)

The subject of the payment that the surrogate receives from the clinic for her work, and wondering how different or similar it is to prostitution, was well understood by Anne, who describes how she deals with it from a place of power, meaning that she has the choice to use her body for sexual education and training for the benefit of the patient.

There are those who look at it as if I am letting my body be taken advantage of, and I say no…I am the master of my body. I help people who don’t know what I know to reach the next stage of development. I am not taken advantage of. I bring myself and I take advantage of my skills to bring about a new approach. On the contrary, I enlarge my power as a woman who can decide to use what is called my body…I can decide to use it from a very, very high and powerful place, not from a low place. It makes a very big improvement. (Annie)

The issue of receiving payment and the similarity and difference of the surrogate practice compared to paid sex with a sex worker was also addressed by the male interviewees:

She said to me something like, “Excuse me, but I am paying for this session so we need to French kiss,” and that is something that should never be said to a surrogate. At that moment I said to her, listen, it seems to me you are confused here about, about the definition of the role and the definition of the profession, so we are going to stop this session now, and you go home and speak with the sex therapist and then we will meet again after it is explained to you very well what a surrogate is and what the difference is between a surrogate and a prostitute…We are not prostitutes and we are not female or male escorts or anything like that, our role is to be your partners… (Dor)

It seems that the issue of payment is not only confusing to the general public and the surrogates themselves but also to the recipients of the treatment at the clinic, who may think that since they are paying for the treatment, they, as service consumers, can also determine what will happen in the meetings in terms of sexual relationships. In reality, there is meant to be a mutual process of connection and creating a relationship that is forged over time. The payment, in the experience and perception of the surrogates, is for the relationship and the ability to connect and be with the other. This is not only in the sexual aspect but also in the many broader aspects of the relationship. Such issues are discussed extensively during the treatment itself. However, it is likely that the different interpretations, anchored in social narratives regarding sex and payment, go through a parallel process for surrogates, their patients, and society. Thus, the internalization of cultural social discourse (about whether a person who is paid for sex is a sex worker or not) affects and structures the surrogates’ perception of professionalism and self-identity.

## Discussion

Our purpose in this paper was to uncover the experiences of SPs. Although the literature does cover the experiences of SPs’ clients (e.g., Aloni et al., 1994, 2007; Bechthold, 2020), very little has been said about SPs’ own experiencesis known about the experiences of the SPs themselves. We wished to shed light onto the SPs themselves, amplifying their voices and sharing their work experiences, while better understanding the boundaries of their work. Our findings suggest that the SPs’ experiences are complex due to the special nature of the treatment and the boundaries required for it. Questions such as "Am I a friend? A lover?" vary from client to client and can even evolve with respect to a given client during treatment. Each SP answers these questions differently and constructs different boundaries accordingly.

According to Goffman (1959), we all wear masks and perform according to the different roles we are expected to play in day-to-day life—moving from the authentic self "behind the scenes," to our social roles on "the front stage.” While negotiating their roles, SPs need to put on various personas and “exit and enter” different stages. Moreover, such transitions in personas take place in their private and social lives, as they also have roles as family members, and as employees in their other occupations. Navigating various roles and moving between stages, all of which require different personas, leads to the creation of relevant boundaries.

In the social and cultural context, questions such as "Given that I get paid for sex, how does that make me different from a sex worker?" often arise. Furthermore, whereas SP treatment is subsidized by Israel’s Ministry of Defense and Ministry of Health, which could serve to legitimate the social discourse regarding SP treatments in Israel, SP clinics require that SP identities remain secret, and that SPs keep their practice confidential. The current social discourse thus presents significant challenges for SPs to establish a professional identity that is out in the open and recognized by the public. In Goffman’s terms, it prevents them from playing their role and putting on their SP persona on the “front stage.” It is reasonable to assume that the demand from surrogates not to reveal their occupation has to do with Israeli society's conservative culture. This may contribute to the confusion between the roles of a sex worker and a surrogate.

From our findings it seems that despite the ambivalent voices SPs experience, they nevertheless manage to conceptualize their own role, as Winnicott (1991) conceptualizes the “potential space” in which the two subjects (in this case, the surrogate and the client) play and communicate with each other but also between each one and their own self. Moreover, there are numerous personas and identities that SPs hold in their personal and professional lives, all of which are constructed through society and culture.

We suggest that the ability to perform as an SP requires a strong sense of self-confidence, flexibility, and high cognitive abilities. The lived experience of surrogates, as it appears from current research, is that most of the time they manage to juggle the various boundaries and roles. Moreover, it seems that the emotional benefits they receive are what encourages and motivates them to continue.

### Limitations

All the interviewees in this study belong to the same clinic—the first and the main clinic established in Israel. We did not have access to other clinics, and we have no information about other surrogate services in Israel. In addition, our sample relates to the Jewish population in Israel only, and we have no information about similar clinics or similar practices in other sectors of Israeli society. As far as is known to us, there are no surrogates in Arab society in Israel or in the ultra-Orthodox community. In addition, this study did not examine the clients’ experiences and how they experience boundary setting by the surrogates. This perspective is of interest and important for further research.

### Conclusions

The theories by Goffman (1959) and Winnicott (1991) were used to suggest that we all, in different degrees, “play” our different personas in various systems in our lives. However, it appears that SP’s transitions from one performance to another are more dramatic, in the sense that their role requires the involvement of sexual and emotional helping-relations with their patients. Moreover, SP are obliged to have secrecy at all levels and in various relationships in their lives: they use different names and identities in the clinic, keeping their personal lives “outside” the clinic doors, and leave their clinic lives behind when they put on their other personas—as family members, partners, in their other occupations and in their social interactions. These transitions between the different personas are challenging, and can often be confusing. It requires them to constantly confront and maneuver within opposed boundaries—rigid and at the same time very loose boundaries. Not just between the various clients, and the various realities inside and outside the clinic, but also within their own selves.

## Appendix 1: Interview Questions

Could you please provide information about yourself, including your profession and how long you've been working as a surrogate? What led you to choose this profession, and what has been your experience like as a surrogate?

We would appreciate it if you could share your insights into the experience of being a surrogate. What does it entail for you, and what aspects do you find fulfilling or challenging about it?

While you may not have experience as a surrogate in other countries, do you believe there are unique challenges specific to the Israeli context? If so, could you elaborate on them? Alternatively, we are interested in hearing your perspective even if you believe there are no unique challenges.

How do your family, romantic partner, friends, and community perceive your profession as a surrogate? Have you encountered any negative experiences in your work that you would like to share, if you are comfortable doing so?

Finally, is there anything else you would like to share with us? Are there any aspects of the surrogate life experience that we haven't addressed but you believe are important to consider?

## Footnotes

1 Retrieved from https://www.dr-aloni.co.il/surrogate_treatment/men_and_women (February 14, 2023).

2 Retrieved from: https://www.dr-aloni.co.il/surrogate_treatment/men_and_women (February 14, 2023).

3 An anonymous reviewer also proposed that one might also consider the notion of counter-transference that may take place in SPs responses to their clients, in particular when they exhibit strong negative responses.
